# Reliability-Oriented Multi-Class Fault-Injection Acceleration for Digital Integrated Circuits via Heterogeneous Spatio-Temporal Graph Learning

**DOI:** 10.3390/mi17091059

**Published:** 2026-09-04

**Authors:** Jiaqi Lu, Changqing Xu, Huixin Peng, Guoxing Zhang, Liang Wang, Xinfang Liao, Yi Liu, Yintang Yang

**Affiliations:** 1 Guangzhou Institute of Technology, Xidian University, Guangzhou 510000, China; 24181214227@stu.xidian.edu.cn (J.L.); liaoxinfang@xidian.edu.cn (X.L.); 2 Faculty of Integrated Circuit, Xidian University, Xi’an 710126, China; yiliu@mail.xidian.edu.cn (Y.L.); ytyang@xidian.edu.cn (Y.Y.); 3 Beijing Microelectronics Technology Institute, Beijing 100076, China; huixinelf@163.com (H.P.); wangliang150200@163.com (L.W.); 4 Information Center of State Administration of Science, Technology and Industry for National Defense, Beijing 100048, China; 5 National Innovation Center of Radiation Application, Beijing 100076, China

**Keywords:** integrated-circuit reliability, single-event upset, fault injection, functional safety, graph neural network, reliability simulation

## Abstract

The pre-silicon reliability analysis of digital integrated circuits relies on fault-injection campaigns to characterize how single-event upsets propagate into distinct system-level outcomes. Exhaustive gate-level injection is expensive, whereas most learning-based accelerators collapse failure manifestations into a single binary label, thereby providing limited support for reliability diagnosis and follow-up analysis. We present a reliability-oriented reduced-campaign framework that predicts the monitor-defined outcome type of flip-flop (FF)-level cases omitted from a fixed circuit/workload campaign. For each circuit, Cadence Xcelium first performs SA0/SA1 screening and retains an FF when at least one stuck-at polarity produces a monitored failure. Within the resulting transient campaign, FeatureCoverage selects FF–time cases using structural attributes and fault-free activity statistics without reading their transient-fault outcomes. HSTGNN then combines a 60-cycle FF logic-value window from the same fault-free waveform, netlist-derived FF–FF topology and gate-path attributes, and module hierarchy to predict five outcomes: C0 No Error, C1 Result Error, C2 Exception Error, C3 Timeout Error, and C4 Safety Error. On the I2C, SPI, FIFO, and RISC-V benchmarks, HSTGNN achieves an 81.2–99.0% macro F1 on more than 38,000 held-out FF–time cases under a 70% labeled fault-injection budget comprising 60% training and 10% validation cases. FeatureCoverage also yields the highest macro F1 across all four label-free split strategies in every evaluated circuit. The proposed framework predicts the remaining 30% of cases while preserving reliability-relevant outcome semantics that binary acceleration discards. The evidence is limited to within-campaign prediction and supports, rather than replaces, quantitative reliability and diagnostic-coverage analysis.

## 1. Introduction

High-reliability digital integrated circuits are required in automotive, avionics, aerospace, and other safety- or mission-critical electronic systems. Transient faults and radiation-induced soft errors can corrupt sequential state and propagate into crashes, safety-monitor violations, incorrect results, or timing failures. Pre-silicon fault injection provides controlled evidence about these effects and supports vulnerability analysis, diagnostic follow-up, and reliability-oriented design decisions. In functional-safety workflows, including ISO 26262 [1] -style analyses, engineers must distinguish fault manifestations rather than merely determine whether an execution differs from its fault-free reference.

The cost of obtaining this evidence is a central obstacle. Gate-level campaigns inject many flip-flops (FFs), repeat injections across time windows and workloads, and simulate long enough to observe architectural or system-level failures. Exhaustive fault injection therefore becomes impractical for large digital designs. Learning-based methods can reduce the campaign volume by predicting fault effects before every candidate fault is injected. Graph neural networks (GNNs) fit this setting because digital circuits contain directed dependencies among FFs, gates, and modules together with workload-dependent activity. Prior SEU-acceleration work uses spatio-temporal graph models, including STGCN-style architectures, to predict binary observable-error labels.

Binary acceleration, however, provides an incomplete interface to reliability analysis. It can separate No Error cases from observable errors, but it merges Result Error, Exception Error, Timeout Error, and Safety Error into one positive class. These outcomes imply different propagation paths, diagnostic priorities, and potential mitigation actions. A reliability-oriented accelerator should reduce the number of simulations without discarding these distinctions. It must also choose which cases to simulate before the outcomes of skipped cases are available; a sampler that uses those labels would not represent an operational reduced-campaign workflow.

We therefore study fixed-campaign, multi-class acceleration as an intelligent reliability-simulation task. The method predicts unexecuted FF–time cases within the SA0/SA1-screened transient campaign of a single circuit/workload and preserves their monitor-defined outcome categories for downstream reliability analysis. We do not claim cross-circuit or cross-workload transfer, a physical radiation-response model, or direct estimation of ISO 26262 diagnostic coverage.

In this paper, we make three main contributions:

- We formulate reliability-oriented, FF-level multi-class fault-injection acceleration as a reduced-campaign prediction task that preserves distinct failure manifestations for subsequent analysis.
- We propose FeatureCoverage, which is a pre-injection sampler that allocates the post-screening transient-fault budget using netlist/hierarchy attributes and fault-free workload activity without reading the transient outcomes of unsimulated cases.
- We design HSTGNN to combine temporal FF activity, netlist topology, and module hierarchy, and we show across four digital circuits that it predicts the remaining 30% of campaign cases with 81.2–99.0% macro F1 under the main 70% labeled budget.

## 2. Related Work

### 2.1. Soft-Error Reliability and Functional-Safety Analysis

Functional-safety analyses, including ISO 26262-based workflows, evaluate electronics through fault effects, safety mechanisms, and diagnostic coverage. Simulation-based fault injection observes controlled transient-fault behavior before silicon. QEFIRA supports ISO 26262 Part 11 digital-component failure modes and logs outcomes for safety metrics [2]. FuSa EDA studies show that diagnostic-coverage estimates vary with tool-level fault-space coverage, so fault classification and traceability cannot be afterthoughts [3]. We keep outcome-type labels that binary acceleration would discard, but we do not replace diagnostic-coverage analysis.

### 2.2. Intelligent Acceleration of Reliability Simulation

Machine learning supports EDA tasks, including test, power, timing, and reliability. In reliability simulation, learned predictors can identify vulnerable state elements or infer fault effects so that a campaign concentrates simulation effort on informative cases. For fault-injection campaign reduction, Lu et al. show that graph predictors exploit circuit structure, VCD-derived activity, and imbalanced fault data better than plain neural networks [4]. Their TCAD work identifies critical FFs using netlist structures, FF features, VCD waveforms, and edge features with GraphSAGE-style models among the highest-performing variants [5]. The closest spatio-temporal baseline, TCAD 2025 STGCN, uses circuit graphs, temporal workload features, and spatial, temporal, and hybrid sampling to reduce simulated SEU injections while maintaining high binary accuracy [6].

Functional-safety graph learning also targets automotive E/E systems. GNN-for-IC surveys cover reliability and security tasks [7]. Das et al. rank faults for selective mitigation [8]; Sun et al. use GAT and explainable GNNs for ISO 26262-oriented critical-node identification and traceability [9,10]. We instead predict multi-class outcomes rather than binary criticality or fault effects, and we use label-free sampling before full labels are available.

### 2.3. Circuit Representation Learning

Circuit representation learning underlies many GNN-based EDA methods. DeepSeq models sequential netlists with temporal gate correlations [11]. DeepGate2 learns functionality-aware gate embeddings from truth-table supervision [12], and DeepGate3 adds transformers and subcircuit modeling for scalability and generalization [13]. Circuit2Graph [14] and HOGA [15] study circuit graph conversion and scalable attention encoders. CircuitNet 2.0 [16], CircuitOps [17], CktGNN [18], MasterRTL [19], and circuit foundation model surveys [20] add datasets and infrastructure for circuit learning. We use these graph representations for monitor-defined outcome prediction under reduced injection budgets and evaluate within-campaign prediction rather than cross-circuit transfer.

### 2.4. Sampling Under Label Scarcity

Fault-injection data are imbalanced: most faults are No Error, while safety-critical outcomes are rare. Offline class balancing can keep class ratios only after labels are known, so it does not, by itself, reduce the injection campaign. Lu et al. introduced spatial sampling over FF locations, temporal sampling over fault-injection times, and their hybrid combination for STGCN-based SEU campaign acceleration [6]. Building on this pre-injection sampling framework, we develop FeatureCoverage to replace purely random case selection with explicit coverage of fault-free activity, synthesis-derived connectivity, and module hierarchy while retaining the same prohibition on using unsimulated transient outcomes. BEC prunes campaigns by classifying bit-level soft-error effects before exhaustive injection [21]. Active learning and coreset methods address label scarcity but often require iterative training or uncertainty estimates.

## 3. Materials and Methods

### 3.1. Reliability-Oriented Fault Injection and Data Definition

**Figure 1:**
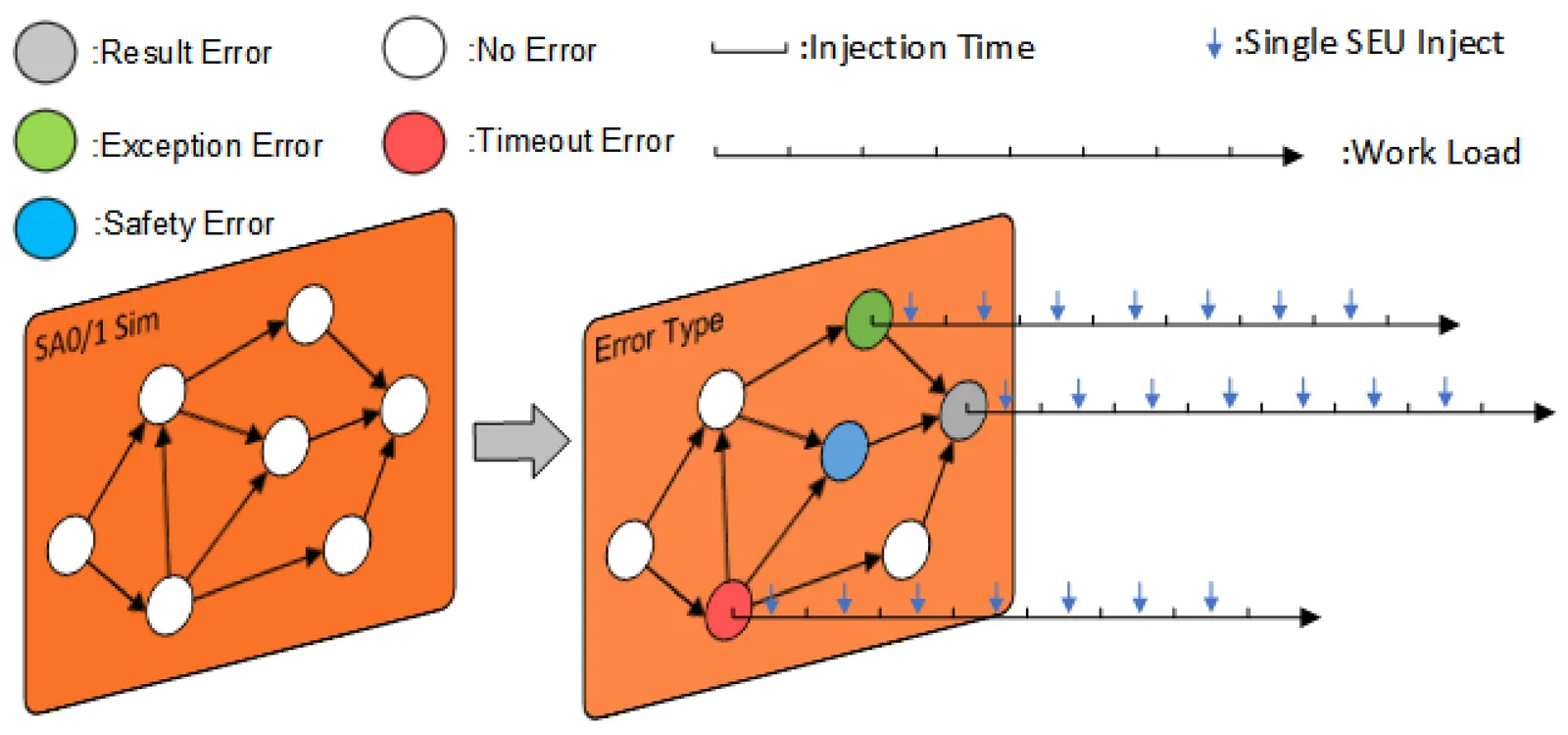
Xcelium fault-injection workflow: SA0/SA1 site screening followed by time-resolved single-SEU injection and monitor-based outcome labeling using C0 No Error, C1 Result Error, C2 Exception Error, C3 Timeout Error, and C4 Safety Error.

The reliability campaign targets single-event upsets at FF nodes. Following prior STGCN-based SEU fault-injection acceleration [6], we treat acceleration as the prediction of unexecuted, post-screening FF–time cases inside a fixed circuit/workload campaign. Executed injections provide ground-truth fault manifestations, while predicted cases extend the outcome map available for subsequent vulnerability triage and diagnostic follow-up. The task is not cross-circuit transfer, cross-workload transfer, physical radiation-response modeling, or diagnostic-coverage estimation. Each graph $G_{k}$ represents one selected transient-injection time $t_{k}$ within the fixed workload rather than a different netlist. All graphs of a circuit share the same SA0/SA1-screened FF namespace, topology, and hierarchy. I2C, SPI, and RISC-V each contain 50 selected injection-time graphs, whereas the FIFO campaign contains 28. Held-out cases therefore come from the same post-screening transient campaign as labeled cases. Their 60-cycle FF windows and activity statistics depend on $t_{k}$ but are extracted only from a separate fault-free execution; the FF–FF edge attributes are netlist-derived structural descriptors rather than activity-derived quantities. Only the monitored outcome depends on the corresponding transient-fault execution. For graph $G_{k}$, the simulator injects one transient bit flip into retained FF $v_{i}$ at $t_{k}$, runs the stimulus, and records $y_{k,i}$. Each supervised sample is therefore an FF–time case $\left(G_{k},t_{k},v_{i},y_{k,i}\right)$, and the complete post-screening transient campaign contains $|V_{\mathrm{cand}}\left|\;\times \;|T\right|$ such cases. Figure 1 shows the data flow.

The data loader reads four tables: nodes with graph_id, node_id, a fault-free temporal window, pre-injection activity/structural attributes, and the post-injection label; graph identifiers for selected injection times; FF–FF connectivity and gate-path attributes; and FF-to-module mappings. These tables define the heterogeneous graph and the supervised labels. Their detailed provenance and use are given below.

### 3.2. Xcelium Fault-Injection Campaign and Label Generation

All gate-level reference, screening, and transient-fault simulations are run with the Cadence Xcelium fault-simulation platform. Xcelium elaborates the synthesized netlist together with the original testbench and uses built-in fault commands, so the RTL and gate-level netlist are not instrumented with fault multiplexers. Every run starts from the same reset sequence and applies the same deterministic workload. Fault activation and candidate transient-injection times are restricted to the active workload interval after reset deassertion. A fault-free run is executed first to record the golden outputs, completion behavior, safety-monitor behavior, and FF VCD waveform used by the feature pipeline.

The campaign then performs site-level screening before constructing the time-expanded transient campaign. For every physical FF $v_{i}\in V_{\mathrm{all}}$, Xcelium executes one stuck-at-0 (SA0) run and one stuck-at-1 (SA1) run with one fault site active per run. The stuck-at value is maintained during the observed workload. The same monitor and golden reference used for the transient campaign determine whether either polarity produces an observable non-C0 outcome. An FF is retained if SA0 or SA1 produces a monitored failure:    

(1) $$V_{\mathrm{cand}}=\left\{v_{i}\in V_{\mathrm{all}}:\;y_{i}^{\mathrm{SA}0}\neq C0\;\vee \;y_{i}^{\mathrm{SA}1}\neq C0\right\}.$$

FFs that remain C0 under both stuck-at polarities are not included as transient-injection sites in the evaluated dataset. Thus, the “FFs” reported in the benchmark table are retained candidate sites rather than the total number of physical FFs in the synthesized circuit.

After site screening, a predefined set of eligible clock-cycle boundaries $T=\{t_{1},\ldots ,t_{K}\}$ is selected from the active workload interval. The selected points exclude reset and terminal intervals and must leave the temporal context and monitor interval required by the campaign. I2C, SPI, and RISC-V each use K=50 injection cycles. Because the FIFO workload has a shorter active cycle sequence, only K=28 workload cycles are selected as transient fault-injection points. The same T is applied to every retained FF of a circuit. For each pair $\left(v_{i},t_{k}\right)\in V_{\mathrm{cand}}\times T$, Xcelium starts from the common reset/workload sequence and injects exactly one SEU through its sequential-gate fault command at $t_{k}$. The command complements the stored value of $v_{i}$ once; the upset is not maintained as a permanent force, and ordinary netlist assignments resume when the FF is next updated. No run contains two simultaneous injected faults.

After injection, simulation continues until normal completion or the campaign timeout. The fixed label encoding is C0 No Error, C1 Result Error, C2 Exception Error, C3 Timeout Error, and C4 Safety Error. When multiple monitor conditions coexist, the deterministic decision priority is Timeout Error, Exception Error, Safety Error, Result Error, and No Error. Each $\left(v_{i},t_{k}\right)$ pair is therefore one independently simulated and labeled case. Table 1 summarizes the complete sequence.

The term “label-free” in FeatureCoverage refers specifically to the target transient-outcome labels $y_{k,i}$: the sampler never reads these labels before selecting transient simulations. The preceding SA0/SA1 screen is a separate, outcome-based site filter. It requires two site-level runs per physical FF but is performed once rather than repeated at every $t_{k}$. The workload counts and acceleration results reported below cover the post-screening transient campaign; they do not include the Xcelium SA0/SA1 screening cost. This distinction bounds the reported simulation reduction and avoids treating the screening stage as a free operation.

### 3.3. Circuit Representation

**Figure 2:**
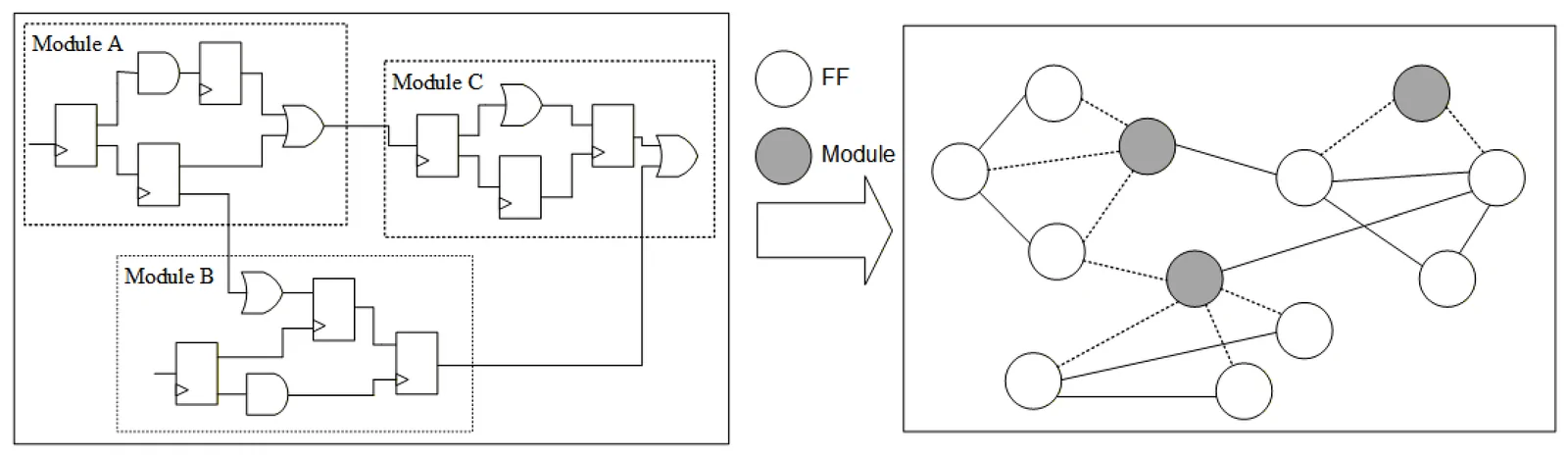
Heterogeneous circuit graph schema with FF topology and module hierarchy.

We represent a sequential circuit as a heterogeneous graph

(2) $$G=(V_{\mathrm{ff}},V_{m},E_{\mathrm{ff}},E_{\mathrm{fm}},E_{\mathrm{mf}}),$$

where $V_{\mathrm{ff}}$ denotes FF nodes and $V_{m}$ denotes module nodes. The edge set $E_{\mathrm{ff}}$ stores FF-to-FF topology. The sets $E_{\mathrm{fm}}$ and $E_{\mathrm{mf}}$ store FF-to-module membership and reverse module-to-FF context edges. Each FF node $v_{i}$ has a fault-free temporal window $x_{i}$, a label-free sampler vector $s_{i}$ containing activity and structural attributes, and, only after its fault-injected execution, a multi-class outcome label $y_{i}$. The HSTGNN consumes $x_{i}$, FF–FF gate-path attributes, and graph/hierarchy relations; $s_{i}$ is used by FeatureCoverage and the tabular controls, but it not used as an additional HSTGNN input. Figure 2 shows the construction.

### 3.4. Feature Provenance and Leakage Control

All workload-dependent inputs are obtained from a separately executed fault-free (golden) simulation. For a candidate injection time, the FF input $x_{i}$ is a 60-cycle logic-value window sliced from the golden VCD waveform. Although the window is aligned with the candidate injection time and may include later golden clock cycles, it contains no injected trajectory: the golden run is completed once before selecting or executing target fault injections. Toggle rate and duty cycle are likewise computed from the fault-free workload trace as the normalized transition frequency and the fraction of sampled cycles at logic one, respectively. This construction follows the fault-free temporal-feature protocol of prior STGCN-based acceleration [6].

The remaining graph inputs are derived from synthesis output and therefore do not require fault simulation. Each FF is a node. Breadth-first search on the synthesized netlist creates an FF–FF edge when the shortest connecting path contains no more than eight combinational gates. The raw eight-position edge vector encodes the type and order of those gates, which are zero padded for shorter paths; label-free PCA reduces it to six components before model training. The module identifier and bidirectional FF–module relations come from the synthesized hierarchy. Table 2 summarizes the complete provenance and point of use.

The preprocessing boundary is enforced as follows. FeatureCoverage receives only the toggle rate, duty cycle, fanin, fanout, and hierarchy ID. HSTGNN receives only the fault-free temporal windows and the synthesis-derived topology, gate-path attributes, and module relations. The outcome column is attached only as a target and is masked to the selected training or validation cases; it is never concatenated with an input feature and is never read by FeatureCoverage. The fully annotated benchmark retains held-out outcomes solely for retrospective scoring after predictions have been produced; those outcomes remain masked during sampling, fitting, checkpoint selection, and inference. For an operationally skipped case, no fault-injected waveform or monitor output is generated or queried. In particular, injected FF values, propagation traces, completion/exception/safety/result monitor signals, and timeout status are excluded from all sampler and model inputs. The graph_id, node_id, and input_coverage_score fields are bookkeeping fields and are not passed as sampler scores or learned features. Consequently, every quantity used to select or predict a skipped case is available without executing that case’s target fault injection.

### 3.5. Multi-Class Fault Outcome Prediction

For node $v_{i}$ in injection-time graph $G_{k}$, the learning target is the categorical monitor outcome $y_{k,i}$. For compactness within a fixed graph, we write this target as $y_{i}$:

(3) $$y_{i}\in \left\{0,1,\ldots ,C-1\right\}.$$

Here, C=5 is the number of outcome classes, and $y_{i}$ is the integer class index assigned by the campaign monitor. Table 3, placed immediately below, defines the fixed integer-to-outcome mapping and monitor semantics. Following common fault-injection outcome practice that separates masked or no-effect executions, incorrect final outputs, detected unrecoverable crashes or hangs, and ISO 26262-oriented diagnostic classifications [22,23,24], C0 denotes a run with no abnormal monitor and golden-reference agreement; C1 denotes completion with an output mismatch; C2 denotes abnormal termination, a crash, or an illegal state before timeout; C3 denotes failure to complete within the campaign timeout bound; and C4 denotes a safety-monitor violation not already classified as timeout or exception. The monitor applies the deterministic priority Timeout Error, Exception Error, Safety Error, Result Error, and No Error when more than one condition is observed.

This mapping is used without permutation throughout the learning pipeline: the integer stored in the dataset is read directly as the training target, output channel *c* represents class C*c*, and the same class index is used for per-class metrics and confusion matrices. Accordingly, C0–C4 must retain the same meanings in every figure, table, and exported result file.

### 3.6. Role in the Reliability-Analysis Workflow

The proposed framework acts as a campaign-acceleration layer between gate-level fault simulation and downstream reliability decisions. First, FeatureCoverage allocates the available injection budget before skipped outcomes are known. Second, HSTGNN learns from the executed injections and assigns outcome categories to the remaining post-screening FF–time cases. The combined observed and predicted outcome map retains separate failure manifestations that engineers can use to prioritize diagnostic replay, vulnerability investigation, or selective mitigation. Prediction fidelity is therefore evaluated across all monitor-defined outcomes rather than only through aggregate binary accuracy.

This role defines the claim boundary of this paper. The predicted categories describe functional manifestations under the implemented workload, monitor, and timeout rule. They do not directly provide a physical soft-error rate, failure-in-time value, radiation cross-section, or ISO 26262 diagnostic-coverage estimate. Such quantitative reliability metrics require a device-level fault model, environmental occurrence rates, and safety-mechanism definitions beyond the scope of this paper. The framework instead reduces the simulation effort needed to construct the multi-class evidence base used by those subsequent analyses.

### 3.7. Fault-Injection Budget Constraint

Let *B* be the fraction of post-screening FF–time cases selected for transient fault injection and labeling before prediction. The selected labels are split into training and validation subsets; the remaining cases receive no target transient injection in the reduced operational campaign and are predicted by HSTGNN. Their stored labels in the fully annotated research dataset are used only for retrospective evaluation. In the main setting, B=70%: 60% for training and 10% for validation and checkpoint selection. Sampling may use only the label-free, pre-injection vector

(4) $$s_{i}=\left[\mathrm{toggle}\_\mathrm{rate},\mathrm{duty}\_\mathrm{cycle},\mathrm{fanin},\mathrm{fanout},\mathrm{hier}\_\mathrm{id}\right],$$

where the first two entries come from the fault-free trace and the remaining entries come from synthesis. FeatureCoverage may access $s_{i}$ but cannot access labels for unsimulated nodes. The HSTGNN itself does not concatenate *S* to its node input; its objective is to train $f_{\theta }\left(G,X\right)$ on selected labeled nodes and predict the remaining outcomes in the same benchmark campaign.

### 3.8. Proposed Method

#### 3.8.1. FeatureCoverage Sampling

Algorithm 1 summarizes the complete FeatureCoverage node-splitting procedure. FeatureCoverage chooses retained FF nodes before the target injections are executed. A purely random split can draw most labeled FFs from a narrow circuit region, leaving feature-similar or hierarchy-related nodes concentrated in either the training or held-out subset. Because skipped-outcome labels are unknown at split time, FeatureCoverage cannot directly balance classes. It instead spreads the injected set across quantities already available from the golden run and synthesized design. Fault-free toggle rate and duty cycle describe workload activity; netlist-derived fanin and fanout approximate local controllability and observability; and the synthesis-derived hierarchy provides module context. The sampler quantizes the four scalar features, groups nodes by hierarchy and feature-bin tuple, covers non-empty groups, and fills any remaining budget from unsampled nodes. It neither reads the temporal input window nor any outcome-dependent quantity when constructing the split.
**Algorithm 1** FeatureCoverage node splitting.**Require:** 
Graphs G; ratios $0<r_{\mathrm{tr}}<1$ and $0\leq r_{\mathrm{val}}<1-r_{\mathrm{tr}}$; features F; group feature *g*; bins *B*; cap *m***Ensure:** 
Training, validation, and prediction dictionaries $S_{\mathrm{tr}},S_{\mathrm{val}},S_{\mathrm{pred}}$  1:**for** each graph G∈G **do**  2:    Let $V=\{v_{1},\ldots ,v_{N}\}$, $N_{\mathrm{tr}}\leftarrow \left\lfloor r_{\mathrm{tr}}N\right\rceil$, and $N_{\mathrm{val}}\leftarrow \left\lfloor r_{\mathrm{val}}N\right\rceil$  3:    **for** each f∈F **do**  4:        Quantize ${\left\{f\left(v_{i}\right)\right\}}_{i=1}^{N}$ into *B* bins and assign bin $b_{i}^{f}$  5:    **end for**  6:    **for** each node $v_{i}\in V$ **do**  7:        Group *i* by key $c_{i}=\left(g\left(v_{i}\right),b_{i}^{f_{1}},\ldots ,b_{i}^{f_{\left|F\right|}}\right)$  8:    **end for**  9:    $I_{\mathrm{tr}}\leftarrow \emptyset$; randomly shuffle all non-empty coverage groups10:    **for** each group *C* **do**11:        Add up to *m* shuffled nodes from *C* to $I_{\mathrm{tr}}$ until $|I_{\mathrm{tr}}|=N_{\mathrm{tr}}$12:    **end for**13:    **if** $|I_{\mathrm{tr}}|<N_{\mathrm{tr}}$ **then**14:        Fill $I_{\mathrm{tr}}$ with shuffled remaining nodes15:    **end if**16:    Shuffle $R=V\setminus I_{\mathrm{tr}}$; assign its first $N_{\mathrm{val}}$ nodes to $I_{\mathrm{val}}$17:    Set $I_{\mathrm{pred}}\leftarrow R\setminus I_{\mathrm{val}}$ and store all original node IDs18:**end for**19:**return** $S_{\mathrm{tr}},S_{\mathrm{val}},S_{\mathrm{pred}}$

#### 3.8.2. HSTGNN: Heterogeneous Spatio-Temporal GNN

**Figure 3:**
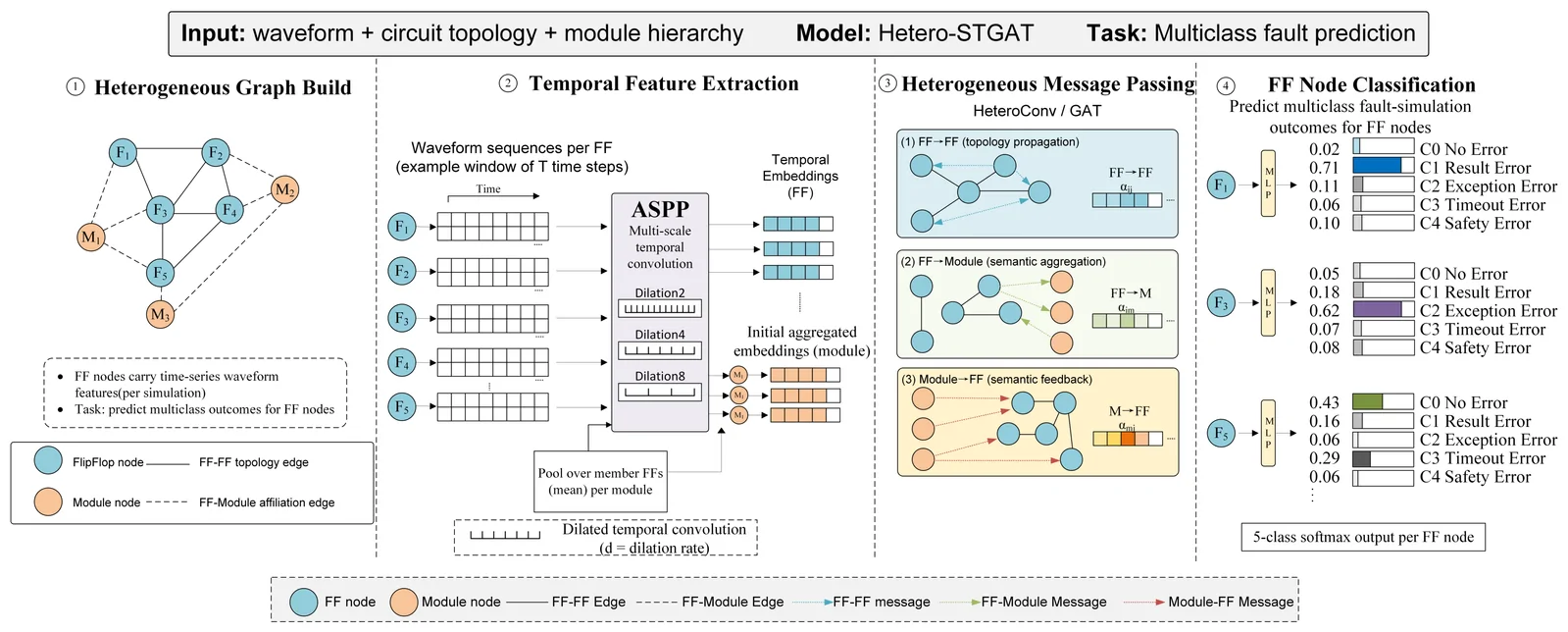
Reliability-oriented HSTGNN pipeline for multi-class FF fault-outcome prediction under a reduced injection budget.

HSTGNN first encodes each FF time window and then applies heterogeneous message passing, as shown in Figure 3. Given FF window $x_{i}\in R^{T}$, ASPP maps it to

(5) $$h_{i}^{\left(0\right)}=\sigma \left({\mathrm{Conv}}_{1\times 1}\left(x_{i}\right)\right).$$

For each d∈D, a dilated temporal convolution extracts

(6) $$h_{i}^{\left(d\right)}=\sigma \left({\mathrm{Conv}}_{\mathrm{temp}}^{\left(d\right)}\left(h_{i}^{\left(0\right)}\right)\right),\;D=\left\{2,4,8\right\}.$$

Concatenating the original and dilated branches gives

(7) $$z_{i}=\sigma \;\left(W_{\mathrm{ASPP}}\;[h_{i}^{\left(0\right)}∥h_{i}^{\left(2\right)}∥h_{i}^{\left(4\right)}∥h_{i}^{\left(8\right)}]\right).$$

Here, $W_{\mathrm{ASPP}}$ is the learnable output-projection matrix that maps the concatenated original and dilated temporal branches to the FF embedding $z_{i}$. Module embeddings use mean-pooled member FF embeddings,

(8) $$z_{m}=\frac{1}{|N_{\mathrm{ff}}\left(m\right)|}\sum_{i\in N_{\mathrm{ff}}\left(m\right)}z_{i}.$$

The FF and module representations then pass through a heterogeneous GAT over FF–FF topology, FF-to-module membership, and module-to-FF context. FF–FF edge features are projected before attention aggregation. The classifier outputs FF-node logits:

(9) $$\hat{Y}=f_{\theta }\left(G,X\right)\in R^{|V_{\mathrm{ff}}|\times C}.$$

#### 3.8.3. Training Objective

Training minimizes circuit-specific weighted cross-entropy on sampled training nodes:

(10) $$L=-\frac{1}{|V_{\mathrm{train}}|}\sum_{i\in V_{\mathrm{train}}}w_{y_{i}}\log p_{\theta }\left(y_{i}|G,X\right),$$

where $V_{\mathrm{train}}$ is the executed set of transient FF–time cases selected for training and $w_{y_{i}}$ is the weight of the target class. The class-weight vectors $\left[w_{0},w_{1},w_{2},w_{3},w_{4}\right]$ are [1,1,1.5,3,1] for I2C, [1,1.5,1,1,1.2] for SPI, [1,1,3,1.5,5] for FIFO, and [1,4,2,1.5,2.5] for RISC-V. These circuit-level vectors are fixed across model seeds and do not depend on validation or held-out labels. Labels outside the training mask are not used by the optimizer.

### 3.9. Experimental Design

#### 3.9.1. Datasets

The benchmark designs are open-source circuits: I2C, SPI, and FIFO are taken from the OpenCores I2C controller, SPI master/slave, and versatile FIFO projects, respectively [25,26,27]; the RISC-V benchmark is based on PULPino [28]. Starting from each original project, we synthesize the RTL, perform FF-level fault injection on the synthesized design, and add monitors to the corresponding testbench to classify outcomes. One Xcelium fault-free execution per workload supplies the VCD-derived node quantities in Table 2; synthesis reports supply topology, gate-path, and hierarchy quantities. Xcelium SA0/SA1 runs then determine the retained FF sites, and the subsequent single-SEU FF–time runs supply the target labels. The data use the four circuit-level tables described above: node features, graph metadata, FF–FF edges, and FF-to-module mappings. From these tables, we construct FF nodes, module nodes, topology edges, and hierarchy edges. Table 4 summarizes the benchmarks.

Each class-distribution total equals the number of retained FFs multiplied by the number of selected injection times; for example, RISC-V contains 2131×50=106,550 independently simulated FF–time cases.

FIFO is highly imbalanced: C4 Safety Error has only 13 samples, and C1 Result Error is absent. Label-free splits can therefore miss rare outcomes during training, making FIFO macro metrics more split-sensitive than those on larger benchmarks.

#### 3.9.2. Compared Settings

We re-implement the three label-free selection modes proposed in Reference [6] as prior-work baselines rather than claiming them as contributions of this paper. Temporal partitions fault-injection times, Spatio samples FF locations within injection-time graphs, and Hybrid combines these temporal and spatial units. On top of this case-selection framework, our FeatureCoverage strategy replaces purely random temporal/spatial case selection with coverage of pre-injection activity, synthesis-derived connectivity, and module hierarchy without accessing unsimulated transient outcomes.

For model comparison, we use a multilayer perceptron (MLP), a feed-forward neural-network baseline trained by back-propagation [29], and a gradient-boosted-tree control, together with GraphSAGE node classification, homogeneous ASPP-STGCN, and HSTGNN with heterogeneous module context. The tabular controls flatten each fault-free FF temporal window together with its pre-injection activity/structural vector, but they do not use graph edges, module relations, node_id, or graph_id. Ablations vary the temporal dilation, spatial depth, and heterogeneous relation types.

We additionally implement a hierarchical HSTGNN control for the severe class imbalance. Its first stage uses all training cases and maps C0 to No Error and C1–C4 to Error. Its second stage uses only C1–C4 cases from the training subset and distinguishes the four error subtypes; the corresponding validation masks are filtered in the same way. At inference, routing uses only the predicted first-stage label: a predicted No Error produces C0, whereas a predicted Error invokes the second-stage C1–C4 output. The true held-out binary label is never used for routing, and performance is computed from the composed five-class predictions on exactly the same held-out mask as the flat model. Both stages use the same FeatureCoverage split, HSTGNN backbone and hyperparameters, input features, and model seeds as the flat model with stage-appropriate output heads. Stage-specific weighted cross-entropy uses $w_{c}=\sqrt{n_{\max}/n_{c}}$ for classes present in that stage’s training set, where $n_{c}$ is the corresponding training count; training uses at most 300 epochs with early-stopping patience 30. The two stages reuse the same executed labels and therefore require no additional fault injections, although they require two separate model-training passes.

#### 3.9.3. Implementation Details

Unless otherwise stated, HSTGNN uses the same model hyperparameters on all benchmarks. The temporal input contains 60 fault-free logic values per FF. The maximum FF–FF gate path is eight positions, and PCA produces the six-dimensional edge attribute actually supplied to the model. ASPP uses dilation rates 2, 4, and 8, and the spatial encoder uses three heterogeneous GAT layers, the hidden size 16, one attention head, and a 4-dimensional edge projection. All compared sampling strategies use a batch size of 10, a learning rate of 0.0005, and a weight decay of 0.00001. The split-strategy study uses ten paired seeds (7, 42, 123, 456, 789, 1024, 2024, 2048, 3407, and 4096); all four samplers use the same seed set. Its uncertainty values are computed over these ten runs. Other model-comparison, ablation, and control results retain their stated three-model-seed protocols. Table 5 lists the default settings.

#### 3.9.4. Evaluation Metrics

Let $TP_{c}$, $FP_{c}$, and $FN_{c}$ denote the number of true positives, false positives, and false negatives for class *c*. We compute class-wise precision, recall, and F1 as

(11) $$P_{c}=\frac{TP_{c}}{TP_{c}+FP_{c}},\;\;\;\;R_{c}=\frac{TP_{c}}{TP_{c}+FN_{c}},\;\;\;\;F1_{c}=\frac{2P_{c}R_{c}}{P_{c}+R_{c}}.$$

For each circuit, let $C_{\mathrm{camp}}$ be the fixed set of classes present in its complete post-screening campaign. The macro F1 is the unweighted mean over this fixed set,

(12) $$F1_{\mathrm{macro}}=\frac{1}{|C_{\mathrm{camp}}|}\sum_{c\in C_{\mathrm{camp}}}F1_{c}.$$

We also report accuracy, macro recall, and per-class F1. In the reliability-oriented interpretation, these metrics quantify how faithfully a reduced campaign preserves the monitor-defined outcome map produced by exhaustive injection; they are not direct physical reliability metrics. Macro and per-class metrics are emphasized because aggregate accuracy can hide failures on rare but reliability-relevant outcomes. If a campaign-present class is absent from an evaluated subset, its precision, recall, and F1 contribution is set to zero rather than silently removing the class from the macro average. A class absent from the complete campaign is undefined and excluded; thus, the fixed sets are C0–C4 for I2C and RISC-V, C0/C1/C3/C4 for SPI, and C0/C2/C3/C4 for FIFO.

For each true error class c∈{1,2,3,4}, we additionally report the false-No-Error rate

(13) $$Q_{c\to 0}=\frac{N(y=c,\hat{y}=0)}{N(y=c)},$$

where $\hat{y}=0$ denotes a C0 No Error prediction. This directional quantity isolates reliability-critical false-benign decisions from confusion among error subtypes. It is undefined when class *c* is absent from the complete circuit campaign. Confusion matrices are row-normalized by true-class support so that each cell represents $N\left(y=c,\hat{y}=j\right)/N\left(y=c\right)$.

## 4. Results

### 4.1. Outcome-Prediction Fidelity Under Reduced Injection Budgets

**Figure 4:**
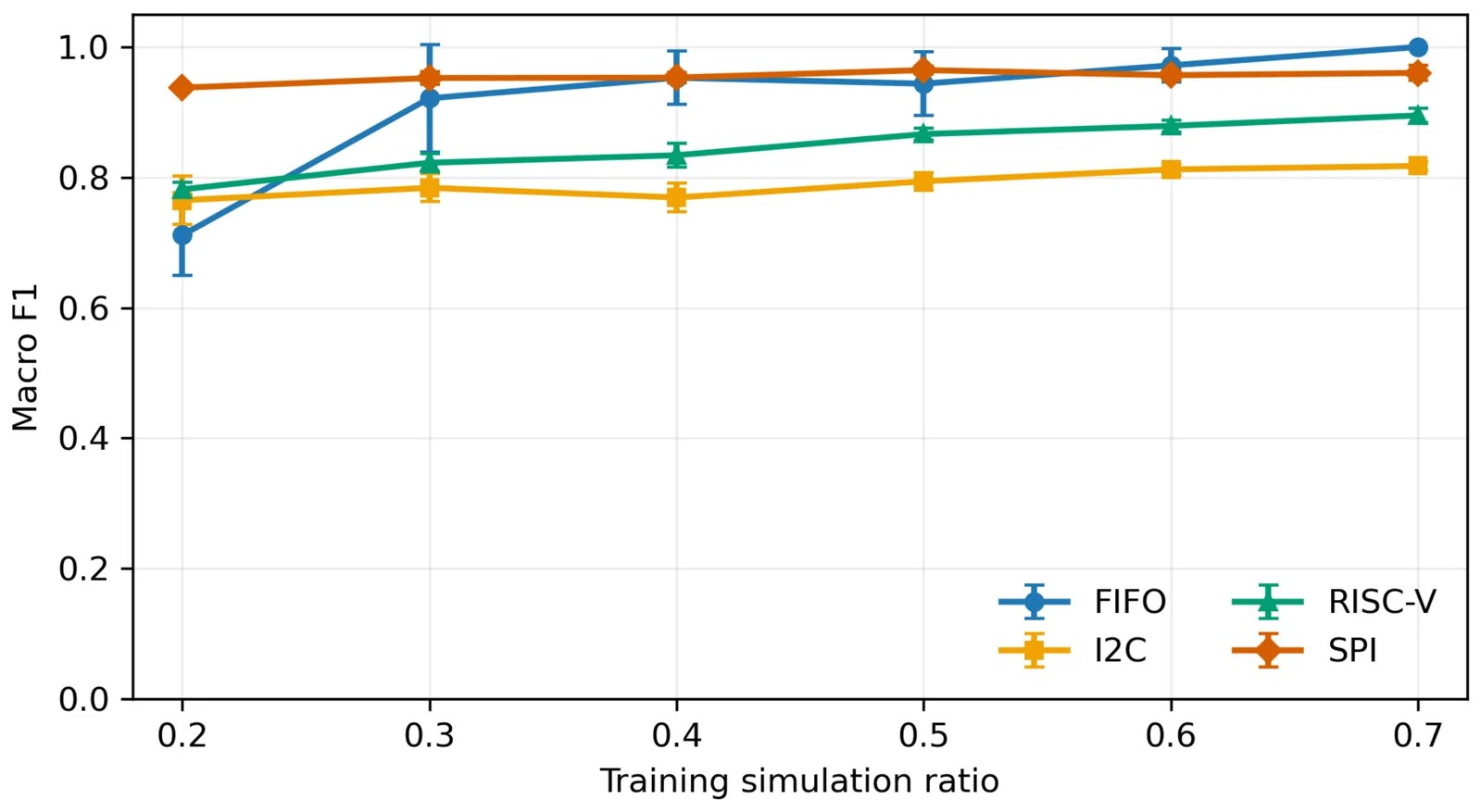
Fidelity of monitor-defined reliability outcomes versus the training-label fraction under FeatureCoverage, measured by macro F1 (mean ± std over seeds).

Figure 4 compares macro F1 across training-label fractions; validation labels are counted separately in the total labeled budget. The main setting uses a 70% labeled budget, split into 60% for training and 10% for validation, with the remaining post-screening FF–time cases used for prediction. Performance remains stable on most benchmarks even below this setting. At a 30% training-label fraction, I2C, SPI, FIFO, and RISC-V reach 78.4 ± 2.1%, 95.2 ± 0.9%, 92.1 ± 8.3%, and 82.2 ± 1.5% macro F1. At 20%, SPI and RISC-V still reach 93.8 ± 0.3% and 78.2 ± 1.0%. FIFO is most sensitive at the smallest budget because rare C4 can be weakly sampled, but it recovers once the training budget reaches 30–40%. Very rare outcomes may still need targeted sampling.

### 4.2. Data Split Strategy Study

To isolate fault-injected-node selection, we run a split-strategy study using the same HSTGNN architecture, loss, and optimizer. Temporal, Spatio, and Hybrid are prior-work baselines that follow the time-, FF-location-, and combined sampling concepts introduced in Reference [6], respectively; they are not sampling contributions of this paper. FeatureCoverage is our extension of this framework and selects cases to cover fault-free activity, synthesis-derived connectivity, and hierarchy rather than relying only on random temporal or spatial selection. The comparison therefore evaluates our proposed coverage criterion against the three sampling strategies of Reference [6] while using only quantities available before target injection.

For this sampler-only comparison, Table 6 uses the same nominal 60%/10%/30% training/validation/prediction protocol and batch size of 10 for all modes; it is separate from the main 70% labeled-budget model comparison reported later. High-variance I2C Temporal and FIFO Spatio runs use at least 300 training epochs. Because Temporal and Hybrid allocate graph/time units whereas Spatio and FeatureCoverage allocate FF cases, integer unit boundaries produce the realized predicted-case counts reported in Table 7; these differences are part of the operational sampler-specific evaluation and prevent interpreting Table 6 as a common-test comparison.

The ten-seed results retain FeatureCoverage as the highest-macro-F1 strategy on all four circuits. Its mean macro F1 and 95% Student-*t* confidence intervals are 81.01% [80.78, 81.24] on I2C, 95.32% [95.27, 95.37] on SPI, 98.85% [98.53, 99.18] on FIFO, and 87.60% [87.05, 88.16] on RISC-V. In paired seed-wise comparisons against the strongest competing mean on each circuit, the FeatureCoverage gains are 3.44 percentage points [2.10, 4.77] over Hybrid on I2C, 2.11 [2.08, 2.13] over Spatio on SPI, 56.28 [52.37, 60.18] over Spatio on FIFO, and 6.36 [6.13, 6.58] over Spatio on RISC-V. These paired intervals quantify repeated-run variation but do not remove sampler-specific test-population differences. We therefore interpret the gains as the compound operational effect of allocating cases between execution and prediction rather than solely as improved learning from a better training set.

Across these ten runs, no class in $C_{\mathrm{camp}}$ is absent from either the executed or predicted subset for any circuit or sampler; the observed absence frequency is therefore 0/10. In particular, FIFO C4 has 9–11 executed cases and 2–4 predicted cases, depending on the sampler. The fixed-set macro calculation nevertheless assigns zero to any campaign-present class should it be absent in another split. Supplementary Table S1 records every circuit–sampler–seed support, and Supplementary Table S2 records all twelve paired FeatureCoverage comparisons with 95% confidence intervals and paired *t*-test results.

### 4.3. Model Ablation

**Figure 5:**
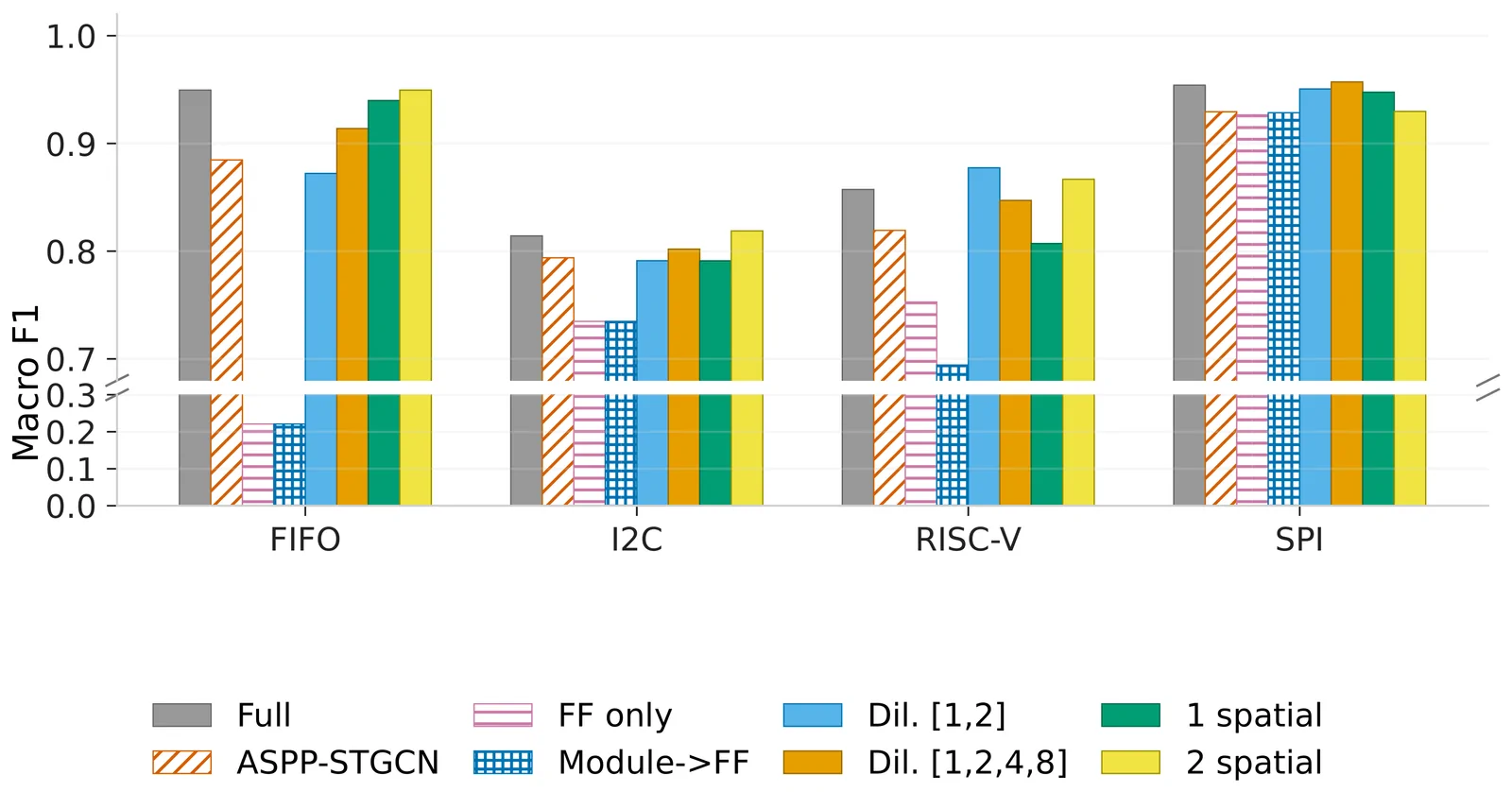
Macro-F1 ablation under the FeatureCoverage protocol.

Figure 5 compares full HSTGNN with reduced variants under the same FeatureCoverage split protocol, validation ratio, loss, seed set, and training schedule. The ablations isolate the heterogeneous FF/module propagation, temporal receptive field size, and spatial message-passing depth. Replacing HSTGNN with homogeneous ASPP-STGCN removes relation-specific FF/module attention and lowers the macro F1 on all four circuits, which suggests that module context adds information beyond FF–FF topology. The FF-only and module-to-FF variants show the same pattern, especially on FIFO and RISC-V, where minority outcomes benefit more from contextual propagation.

Temporal variants test whether the default dilation set [2,4,8] is needed. Shorter dilation [1,2] and denser dilation [1,2,4,8] can be competitive on individual circuits, but the default is most consistent across the evaluated campaigns. Spatial-depth variants show a similar pattern: one heterogeneous layer often under-propagates structural context, while two layers recover most performance, and three layers give the default full model. FIFO ablations should be read with the rare-class caveat that reduced variants can fail when tiny C4 is absent or weakly represented in simulated training labels.

### 4.4. Comparison with Learning-Based Baselines

We compare HSTGNN with feature-only and graph-based baselines. The tabular MLP and histogram gradient-boosted tree (HistGBDT) baselines test whether flattened fault-free FF temporal windows and the pre-injection sampler attributes alone explain the results; they exclude graph edges, module relations, node_id, and graph_id. GraphSAGE follows the TCAD 2024 critical-FF work [5], and STGCN follows TCAD 2025 [6]. Table 8 uses the same 70% executed-label budget as the main protocol: 60% training, 10% validation, and 30% held-out post-screening FF–time cases under the same campaign split.

Binary GNN baselines are adapted with a five-class head and multi-class training. Within the baseline-comparison protocol, all methods use the same labels, benchmarks, FeatureCoverage split protocol, and training budget. Macro F1 is the primary metric because accuracy can hide minority-outcome failures.

Table 8 shows that feature-only tabular baselines are substantially weaker than graph predictors on most circuits, especially SPI, FIFO, and RISC-V. GraphSAGE and STGCN improve over tabular controls but preserve minority outcomes less faithfully because they lack the full combination of multi-scale temporal encoding, FF topology, and module hierarchy. HSTGNN gives the highest macro-F1 on all four circuits. FIFO remains sensitive to whether rare C4 cases appear in the training split.

Table 9 gives the per-class F1 results under the main 70% labeled FeatureCoverage budget: 60% training and 10% validation with remaining cases held out for prediction. The model learns dominant C0 and still recovers most of the monitored error outcomes. SPI reaches 95.34 ± 0.14% macro F1 and FIFO 98.98 ± 0.94%. FIFO should not be over-interpreted, however, because the complete campaign contains only 13 C4 Safety Error cases, the held-out partition contains only two C4 cases, and C1 is absent from the complete FIFO campaign. RISC-V is harder on minority C1 Result Error and C4 Safety Error but reaches an 88.02 ± 1.53% macro F1. I2C is hardest on C3 Timeout Error, with 58.67 ± 3.15% F1, which points to targeted simulation or calibration as a next step. These results indicate that the evaluated labels are sufficiently predictable to retain outcome-type information that binary prediction would collapse.

### 4.5. Identity-Aware and Reliability-Directional Controls

Because the main within-campaign protocol can place different injection times of the same physical FF in the executed and predicted subsets, we compare HSTGNN with two training-label-only identity controls. The per-FF historical-majority control assigns each held-out case the most frequent training outcome previously observed for that physical FF. The module-majority control uses the most frequent training outcome in the corresponding module. Both controls fall back to the global training-set majority when no matching training history exists; ties are resolved first by global training frequency and then by class index. No validation or held-out outcome is used by either lookup rule. Table 10 shows that any repeated FF identity contains useful campaign-specific information, especially on I2C and SPI, but does not reproduce the full HSTGNN result. Because node_id and graph_id are not HSTGNN inputs, the comparison tests how far direct identity lookup alone can go rather than adding another feature to HSTGNN. This finding also reinforces the transductive claim boundary: the primary task predicts unsimulated FF–time cases within a partially executed fixed campaign rather than outcomes of wholly unseen physical FFs.

The composed hierarchical baseline reaches 79.00%, 94.11%, 49.62%, and 86.59% macro F1 scores on I2C, SPI, FIFO, and RISC-V, respectively, and it does not outperform the flat weighted model on any circuit. The differences are modest on I2C, SPI, and RISC-V, but FIFO exposes a failure mode hidden by its 94.58 ± 0.94% hierarchical accuracy: its macro F1 is only 49.62 ± 4.88% with 5.80% C2 recall and 0% C4 recall. The second stage has very little evidence for these rare FIFO subtypes (60 C2 and 13 C4 cases in the complete campaign), so separating error detection from subtype prediction does not by itself resolve the imbalance. I2C C3 also has a 22.73% false-No-Error rate under the hierarchy compared with 14.39% for the flat model.

We therefore retain the flat weighted HSTGNN as the main predictor and treat the hierarchy as an informative negative control rather than evidence that two-stage decomposition is generally advantageous.

**Figure 6:**
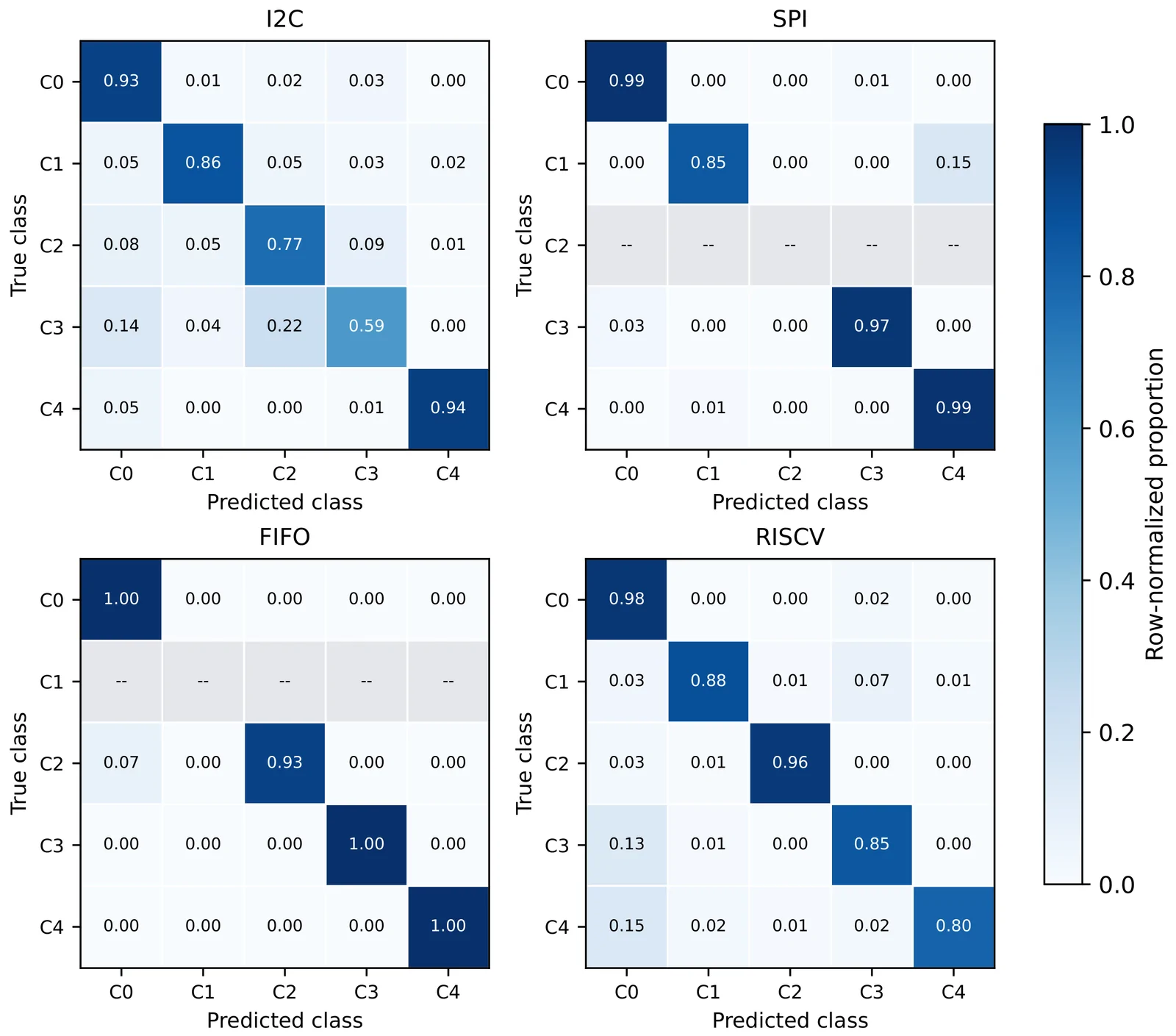
Row-normalized confusion matrices of the flat weighted HSTGNN under the final 60%/10%/30% FeatureCoverage protocol, which is pooled over three model seeds. Rows are true classes and columns are predicted classes; “–” denotes a class absent from the complete circuit campaign.

Aggregate F1 does not distinguish a wrong error subtype from a reliability-critical false-benign decision. We therefore report per-class recall together with the fraction of each true error class predicted as C0 No Error. Table 11 and Figure 6 use predictions from the same final three-seed flat weighted HSTGNN protocol. Recall and false-No-Error rate are reported separately because the remaining errors are confusions among C1–C4 rather than C0 predictions. The largest false-No-Error rates occur for I2C C3 Timeout Error (14.39%), RISC-V C3 Timeout Error (13.36%), and RISC-V C4 Safety Error (14.72%). These cases should be prioritized for confidence-triggered Xcelium replay rather than suppressed solely by the learned prediction.

Table 12 reports the post-SA-screening transient fault-injection workload. The graph model does not change the cost of one Xcelium gate-level run; it reduces the number of FF–time pairs that must receive the transient SEU injection. The executed-case count includes 60% training and 10% validation labels. For RISC-V, the complete post-screening transient campaign contains 106,550 FF–time cases. The 70% labeled FeatureCoverage setting executes 74,600 transient injections, trains in 38.90 min, and predicts the 31,950 remaining outcomes. As stated above, these counts and times exclude the preceding two-polarity SA0/SA1 site-screening runs.

## 5. Discussion

### 5.1. Implications for Integrated-Circuit Reliability Analysis

Multi-class prediction retains outcome types that binary labels would merge, and these labels are predictable in the evaluated campaigns. In particular, the main 70% labeled-budget setting predicts the remaining 30% of post-screening FF–time cases while retaining separate exception, safety-monitor, result-mismatch, and timeout outcomes. This distinction is useful for a pre-silicon reliability analysis of digital integrated circuits because different failure manifestations motivate different diagnostic follow-up and mitigation priorities. The method therefore improves the efficiency of evidence generation without redefining the reliability quantities computed downstream. The predicted classes are monitor-defined outcomes and must not be interpreted as direct estimates of soft-error rate, radiation cross-section, or ISO 26262 diagnostic coverage.

### 5.2. Effect of Sampling and Heterogeneous Modeling

FeatureCoverage gives the highest macro F1 score among the Temporal, Spatio, Hybrid, and FeatureCoverage strategies on all four circuits. Unlike class-balanced sampling performed after simulation, FeatureCoverage does not read the labels of skipped cases. Its advantage is therefore consistent with the operational constraint of a reduced fault-injection campaign: the cases to simulate must be chosen before the remaining outcomes are known. The largest gains occur on FIFO and RISC-V, for which the coverage of fault-free activity, netlist connectivity, and hierarchy helps avoid training sets concentrated in narrow temporal or spatial regions.

Heterogeneous spatio-temporal modeling combines information that the evaluated baselines treat separately. Multi-scale temporal convolutions encode workload-dependent FF activity, FF–FF edges represent netlist dependencies, and FF–module relations propagate hierarchy context. The ablations and baseline comparison consistently favor the full HSTGNN, suggesting that module context adds useful information beyond a homogeneous circuit graph. The experiments do not isolate a single universally optimal temporal dilation or graph depth; rather, the default configuration is the most consistent across the four campaigns.

### 5.3. Application to High-Reliability Integrated-Circuit Workflows

Within a high-reliability design flow, the proposed method can serve as an intelligent triage layer for expensive gate-level simulations. Executed injections establish the campaign-specific training evidence, predicted outcome types extend coverage to skipped post-screening FF–time cases, and the resulting multi-class map can guide detailed replay or mitigation studies toward exception, safety-monitor, result-mismatch, and timeout manifestations. This positioning is relevant to digital systems intended for automotive, avionics, and aerospace applications, where reliability analysis must distinguish failure consequences under constrained verification budgets.

The current model should nevertheless be used as an analysis aid rather than an autonomous certification filter. Because individual predictions are not calibrated and rare outcomes remain split-sensitive, safety-critical cases should not be discarded solely on the basis of a predicted No Error label. A deployment-oriented extension should attach calibrated confidence to each prediction and return uncertain or high-consequence cases to gate-level simulation. Such a simulate-or-predict policy would connect campaign acceleration more directly to conservative reliability assurance.

A broader deployment path is a predict–explain–corroborate workflow, following the general engineering pattern of combining data-driven prediction, explainable analysis, and independent multiphysical evidence [30]. In the prediction stage, HSTGNN would rank unexecuted FF–time cases and flag uncertain or high-consequence outcomes. In the explanation stage, temporal attribution over the fault-free logic window, topology/subgraph attribution over FF–FF paths, and module-level attribution could identify the evidence driving an individual classification. In the corroboration stage, cases predicted as error-prone, uncertain, or consequential would be replayed in Xcelium and, where appropriate, checked against radiation-aware device/cell models or an independent quantitative reliability analysis before influencing simulation suppression or mitigation decisions. This paper implements only the prediction stage; it does not claim that these future explanations are causal or that physical corroboration has already been performed.

### 5.4. Limitations and Threats to Validity

The evidence is restricted to within-campaign prediction. Training and held-out cases share a circuit and campaign definition, so the reported results do not establish cross-circuit, cross-workload, or technology-node transfer. The four benchmarks are open-source digital designs and do not represent the full diversity of industrial microelectronic systems. In addition, outcome labels depend on the implemented monitors, timeout bound, golden reference, and deterministic priority rule; changing these definitions can change the learning task. The evaluated population is also conditioned on SA0/SA1 screening: FFs that are C0 under both stuck-at polarities are outside the transient-prediction task. Consequently, the reported accuracy and workload reduction apply to the retained candidate set and should not be interpreted as coverage of every physical FF. The reported transient campaign time excludes the one-time two-polarity screening overhead.

Rare classes remain the principal statistical limitation. FIFO contains only 13 C4 cases and no C1 cases, so even the ten-seed sampler study evaluates C4 on only two to four predicted cases per run. Macro F1 is therefore more informative than accuracy under class imbalance but remains sensitive to individual rare cases. The non-trivial per-FF historical-majority results also confirm that repeated physical-FF identity contributes campaign-specific predictive information. HSTGNN exceeds this direct lookup control, but that gap does not establish generalization to wholly unseen FFs; a grouped-by-FF protocol is a separate, stricter inductive task. The split-strategy study uses ten paired seeds and reports 95% confidence intervals, whereas the model-comparison, hierarchical-control, and directional-error experiments retain three model seeds. The sampler-specific held-out populations and their non-identical realized case counts mean that the split-strategy gains represent a compound case-allocation and prediction effect rather than a common-test estimate of training-set quality alone. The experiments do not establish calibrated uncertainty for individual predictions. Finally, this paper reduces the number of simulated post-screening transient FF–time cases; it does not reduce the cost of an individual gate-level injection or validate predictions through physical radiation testing. Cross-campaign transfer, rare-outcome calibration, adaptive sampling, larger industrial designs, and integration with quantitative diagnostic-coverage analysis remain priorities for future work.

## 6. Conclusions

This paper addresses a key limitation of intelligent fault-injection acceleration for integrated-circuit reliability analysis: binary prediction can reduce simulation cost, but it loses the outcome semantics needed for vulnerability triage, diagnosis, and functional-safety follow-up. We formulate reliability-oriented, reduced-campaign five-class prediction over the Xcelium post-SA-screening FF–time space so that skipped transient gate-level injections retain monitor-defined error categories within fixed circuit/workload campaigns.

Within the SA0/SA1-retained candidate set, FeatureCoverage improves the use of the labeled injection budget by selecting FF–time cases from fault-free activity and synthesis-derived connectivity and hierarchy before target transient injections are executed or skipped transient labels are known, achieving the highest macro F1 among the evaluated split strategies. HSTGNN combines only the fault-free FF window with netlist-derived FF topology, gate-path attributes, and module hierarchy; no injected trajectory or monitor response is used as an input. Under a 70% labeled budget, it achieves 81.2%, 95.3%, 99.0%, and 88.0% macro F1 scores on I2C, SPI, FIFO, and RISC-V, respectively, while predicting the remaining 30% of post-screening FF–time cases. In the baseline-comparison protocol, it also outperforms feature-only controls, GraphSAGE, and STGCN-style baselines on all evaluated circuits.

Together, these results show a practical route to reduce the simulation workload used to generate multi-class soft-error evidence for digital integrated circuits without collapsing fault-outcome types. The method supports subsequent reliability analysis but does not directly estimate physical soft-error rates or diagnostic coverage. The current evidence remains within-campaign; cross-campaign transfer, rare-outcome calibration, confidence-triggered simulation, radiation-aware fault models, and ISO 26262 diagnostic-coverage integration remain future work.

## Figures and Tables

**Table 1:** Xcelium fault-injection and dataset-generation stages.

Stage	Operation	Output
Netlist setup	Elaborate the synthesized netlist and original testbench in Xcelium; execute the normal reset/initialization sequence	Reproducible gate-level testbench
Golden run	Run the fault-free workload once and record reference outputs, monitor states, completion, and FF VCD	Golden reference and label-free activity
SA screening	For each physical FF, run SA0 and SA1 separately; retain the site if either polarity produces a non-C0 monitored outcome	Candidate set $V_{\mathrm{cand}}$
Time selection	Select valid post-reset clock-cycle boundaries with the required temporal and observation interval	Injection-time set T
Transient injection	For every $\left(v_{i},t_{k}\right)$, execute a separate Xcelium run with one state inversion at $t_{k}$ and no concurrent fault	$|V_{\mathrm{cand}}\left|\;\times \;|T\right|$ transient runs
Label/export	Apply the deterministic monitor priority and export node, graph-time, edge, hierarchy, and outcome tables	Multi-class FF–time dataset

**Table 2:** Feature provenance, construction, availability, and use.

Quantity	Dimension	Source and Calculation	Availability	Used by
FF window $x_{i}$	60	Logic values in the candidate-time-aligned window of the fault-free VCD	After one golden run; before target injection	HSTGNN and tabular controls
Toggle rate, duty cycle	2	Transition frequency and high-state fraction from the fault-free workload trace	After the golden run; before target injection	FeatureCoverage and tabular controls
Fanin, fanout	2	Local connectivity counts parsed from the synthesized netlist/report	After synthesis	FeatureCoverage and tabular controls
FF–FF adjacency	—	Shortest FF-to-FF paths with at most eight intervening combinational gates	After synthesis	HSTGNN
Gate-path edge attribute	6	Eight-position gate-type/order vector, zero padded, then reduced to six label-free PCA components	After synthesis and preprocessing	HSTGNN
Hierarchy and membership	1 ID + relations	FF module identifier and bidirectional FF–module relations parsed from hierarchy	After synthesis	FeatureCoverage (ID); HSTGNN (relations)
Module embedding $z_{m}$	16	Mean of encoded temporal embeddings of the FFs assigned to module *m*	Derived from fault-free inputs during inference	HSTGNN
Outcome $y_{i}$	1	Deterministic classification of the target fault-injected run by completion, exception, safety, result, and timeout monitors	Only after that injection is executed	Supervised loss/validation and offline metrics only

**Table 3:** Fixed C0–C4 mapping of the monitor-defined fault outcomes used for five-class prediction.

ID	Outcome	Monitor Definition
C0	No Error	Completes within the bound and matches the golden reference
C1	Result Error	Completes but produces an output that differs from the golden reference
C2	Exception Error	Abnormal termination, crash, or illegal state before timeout
C3	Timeout Error	Does not complete within the campaign timeout bound
C4	Safety Error	Safety-monitor violation not already classified as timeout or exception

**Table 4:** Benchmark statistics and C0–C4 class distributions for the post-SA-screening transient campaigns.

Circuit	Injection Times	Retained FFs	Edges	[C0, C1, C2, C3, C4]
I2C	50	153	14,938	[3631, 1795, 892, 732, 600]
SPI	50	229	22,394	[5654, 206, 0, 4883, 707]
FIFO	28	60	5940	[1304, 0, 60, 303, 13]
RISC-V	50	2131	210,170	[87,821, 1256, 4144, 11,220, 2109]

**Table 5:** Default HSTGNN model and training hyperparameters.

Hyperparameter	Value	Hyperparameter	Value
Classes	5	Time window	60
Raw edge path	8	PCA edge dim.	6
ASPP dilation	[2, 4, 8]	ASPP output	1
Spatial hidden	16	Spatial layers	3
GAT heads	1	Edge hidden	4
Batch size	10	Learning rate	0.0005
Weight decay	0.00001		

**Table 6:** Split strategy comparison with HSTGNN under the nominal 60%/10%/30% training/validation/prediction protocol, mean ± std over ten paired seeds (%).

Circuit	Split	Accuracy	Macro Recall	Macro F1
I2C	Temporal	76.41 ± 2.16	68.50 ± 3.40	66.99 ± 3.10
	Spatio	80.67 ± 3.31	73.48 ± 6.80	72.43 ± 7.61
	Hybrid	83.98 ± 1.43	77.38 ± 1.76	77.57 ± 1.85
	FeatureCoverage	86.96 ± 0.13	81.68 ± 0.40	81.01 ± 0.32
SPI	Temporal	86.84 ± 5.92	78.94 ± 14.79	81.73 ± 16.07
	Spatio	95.89 ± 0.39	93.35 ± 0.19	93.21 ± 0.08
	Hybrid	90.81 ± 1.74	92.45 ± 0.89	92.31 ± 0.81
	FeatureCoverage	97.62 ± 0.10	94.32 ± 0.33	95.32 ± 0.07
FIFO	Temporal	76.28 ± 0.11	25.00 ± 0.00	21.64 ± 0.02
	Spatio	89.11 ± 2.19	40.84 ± 5.98	42.58 ± 5.85
	Hybrid	80.94 ± 0.06	25.00 ± 0.00	22.37 ± 0.01
	FeatureCoverage	99.62 ± 0.15	97.93 ± 0.80	98.85 ± 0.45
RISC-V	Temporal	82.92 ± 0.07	20.00 ± 0.00	18.13 ± 0.01
	Spatio	93.14 ± 0.46	83.65 ± 1.58	81.24 ± 1.08
	Hybrid	88.87 ± 2.33	60.06 ± 14.35	60.60 ± 15.08
	FeatureCoverage	95.67 ± 0.25	89.07 ± 0.69	87.60 ± 0.78

**Table 7:** Class-support ranges across all four samplers and ten paired seeds. “–” denotes a class absent from the complete campaign. Exact sampler–seed supports are provided in Supplementary Table S1.

Circuit	Subset	C0	C1	C2	C3	C4
I2C	Executed	2327–2583	1148–1295	578–661	434–557	375–430
	Predicted	1048–1304	500–647	231–314	175–298	170–225
SPI	Executed	3831–4015	141–154	–	3360–3544	486–501
	Predicted	1639–1823	52–65	–	1339–1523	206–221
FIFO	Executed	891–934	–	37–54	194–219	9–11
	Predicted	370–413	–	6–23	84–109	2–4
RISC-V	Executed	61,287–62,227	888–960	2851–2982	7821–7939	1429–1568
	Predicted	25,594–26,534	296–368	1162–1293	3281–3399	541–680

**Table 8:** Baseline macro-F1 under the 60%/10%/30% split, mean ± std (%).

Circuit	MLP	HistGBDT/MLP	GraphSAGE	STGCN	HSTGNN
I2C	68.77 ± 1.34	70.91 ± 0.50	78.28 ± 0.40	72.06 ± 2.36	81.38 ± 0.67
SPI	52.80 ± 2.91	53.19 ± 0.98	73.78 ± 0.98	66.18 ± 10.19	95.47 ± 1.10
FIFO	22.81 ± 2.80	38.37 ± 6.15	68.03 ± 5.44	25.99 ± 2.59	92.62 ± 9.96
RISC-V	27.04 ± 18.18	33.99 ± 0.34	46.77 ± 1.13	63.70 ± 7.24	88.35 ± 1.76

The second tabular column is HistGBDT for I2C/SPI/FIFO and weighted Torch-MLP for RISC-V.

**Table 9:** Per-class F1 under the 70% labeled budget, mean ± std (%).

Circuit	C0	C1	C2	C3	C4	Macro F1
I2C	92.64 ± 0.33	89.55 ± 0.43	70.77 ± 0.68	58.67 ± 3.15	94.41 ± 0.12	81.21 ± 0.61
SPI	98.11 ± 0.19	88.59 ± 0.56	–	97.60 ± 0.22	97.07 ± 0.34	95.34 ± 0.14
FIFO	99.78 ± 0.21	–	96.16 ± 3.54	100.00 ± 0.00	100.00 ± 0.00	98.98 ± 0.94
RISC-V	97.66 ± 0.28	81.94 ± 2.70	95.60 ± 0.96	84.99 ± 1.90	79.93 ± 2.17	88.02 ± 1.53

C1 is absent from the complete FIFO campaign. The FIFO held-out partition contains 371 C0, 23 C2, 108 C3, and only 2 C4 cases. A class absent from the complete campaign is excluded from the circuit-level macro average.

**Table 10:** Identity-aware and hierarchical controls under the 60%/10%/30% FeatureCoverage split. Values are macro F1 (%).

Circuit	Per-FF Majority	Module Majority	Hierarchical	Flat Weighted
I2C	72.95	12.72	79.00 ± 1.22	81.21 ± 0.61
SPI	76.13	30.83	94.11 ± 1.57	95.34 ± 0.14
FIFO	43.65	42.85	49.62 ± 4.88	98.98 ± 0.94
RISC-V	55.20	25.35	86.59 ± 0.24	88.02 ± 1.53

The two majority controls are deterministic on the fixed split and use only training labels. Hierarchical and flat weighted HSTGNN values are mean ± std over three model seeds.

**Table 11:** Per-error-class recall and false-No-Error rate under the main protocol. Each entry is recall/percentage predicted as C0, which is averaged over three model seeds (%).

Circuit	C1	C2	C3	C4
I2C	85.97/4.77	76.58/8.48	59.09/14.39	94.34/4.59
SPI	84.62/0.00	–	96.94/2.95	98.55/0.00
FIFO	–	92.75/7.25	100.00/0.00	100.00/0.00
RISC-V	87.81/3.36	96.23/2.57	85.01/13.36	80.41/14.72

C2 is absent from the complete SPI campaign, and C1 is absent from the complete FIFO campaign.

**Table 12:** Post-screening transient reliability-simulation workload under the 70% labeled FeatureCoverage budget.

Circuit	Full SEU Cases	Executed Cases (Training + Validation)	Predicted Cases	Test Simulation (ns)	Fault Simulation (min)	Training (min)
I2C	7650	5350	2300	868,140	220.57	9.75
SPI	11,450	8000	3450	7036	330.14	14.87
FIFO	1680	1176	504	1995	74.89	5.74
RISC-V	106,550	74,600	31,950	952,180	3001.16	38.90

## Data Availability

The original contributions presented in this study are included in the article and Supplementary Materials. Further inquiries can be directed to the corresponding authors.

## Supplementary Materials

The following supporting information can be downloaded at https://www.mdpi.com/article/10.3390/mi17091059/s1: Table S1, class-support counts and fractions for every circuit, sampling strategy, seed, phase, and outcome class; Table S2, paired comparisons of the fixed macro F1 between FeatureCoverage and the Temporal, Spatio, and Hybrid sampling strategies.

## Abbreviations

The following abbreviations are used in this manuscript:

ASPP	Atrous spatial pyramid pooling
EDA	Electronic design automation
FF	Flip-flop
GAT	Graph attention network
GNN	Graph neural network
HSTGNN	Heterogeneous spatio-temporal graph neural network
SEU	Single-event upset
STGCN	Spatio-temporal graph convolutional network

## References

[1] (2018). Road Vehicles—Functional Safety.

[2] Almeida R., Silva V., Cabral J. (2024). Virtualized Fault Injection Framework for ISO 26262-Compliant Digital Component Hardware Faults. Electronics.

[3] Bhowmik A., Babukutty S., Taouil M., Fieback M. A Unified Functional Safety EDA Framework for Accurate Diagnostic Coverage Estimation. Proceedings of the IFIP/IEEE International Conference on Very Large Scale Integration (VLSI-SoC).

[4] Lu L., Chen J., Ulbricht M., Krstic M. (2024). Machine Learning Methodologies to Predict the Results of Simulation-Based Fault Injection. IEEE Trans. Circuits Syst. I Reg. Pap..

[5] Lu L., Chen J., Ulbricht M., Krstic M. (2024). Toward Critical Flip-Flop Identification for Soft-Error Tolerance With Graph Neural Networks. IEEE Trans. Comput.-Aided Des. Integr. Circuits Syst..

[6] Lu L., Chen J., Balakrishnan A., Ulbricht M., Krstic M. (2025). Accelerate SEU Simulation-Based Fault Injection With Spatio-Temporal Graph Convolutional Networks. IEEE Trans. Comput.-Aided Des. Integr. Circuits Syst..

[7] Alrahis L., Knechtel J., Sinanoglu O. Graph Neural Networks: A Powerful and Versatile Tool for Advancing Design, Reliability, and Security of ICs. Proceedings of the Asia and South Pacific Design Automation Conference (ASP-DAC).

[8] Das S., Kundu S., Madhusoodhanan P., Pillai P.V., Parekhji R., Raha A., Banerjee S., Natarajan S., Basu K. Graph Learning-Based Fault Criticality Analysis for Enhancing Functional Safety of E/E Systems. Proceedings of the ACM/IEEE Design Automation Conference (DAC).

[9] Sun Y., Huang J., Liao X., Wang Z., Liang L. Graph Attention Networks Based Fault Prediction Framework for Functional Safety Verification. Proceedings of the IEEE International Test Conference (ITC).

[10] Sun Y., Huang J., Liao X., Wang Z., Liang L. ISO 26262-Aligned Functional Safety Verification Framework with Explainable Graph Neural Network. Proceedings of the IEEE/ACM International Conference on Computer-Aided Design (ICCAD).

[11] Khan S., Shi Z., Li M., Xu Q. (2023). DeepSeq: Deep Sequential Circuit Learning. arXiv.

[12] Shi Z., Pan H., Khan S., Li M., Liu Y., Huang J., Zhen H.L., Yuan M., Chu Z., Xu Q. DeepGate2: Functionality-Aware Circuit Representation Learning. Proceedings of the IEEE/ACM International Conference on Computer-Aided Design (ICCAD).

[13] Shi Z., Zheng Z., Khan S., Zhong J., Li M., Xu Q. (2024). DeepGate3: Towards Scalable Circuit Representation Learning. arXiv.

[14] Yamakaji Y., Shouno H., Fukushima K. (2024). Circuit2Graph: Circuits With Graph Neural Networks. IEEE Access.

[15] Deng C., Yue Z., Yu C., Sarar G., Carey R., Jain R., Zhang Z. Less Is More: Hop-Wise Graph Attention for Scalable and Generalizable Learning on Circuits. Proceedings of the ACM/IEEE Design Automation Conference (DAC).

[16] Jiang X., Chai Z., Zhao Y., Lin Y., Wang R., Huang R. CircuitNet 2.0: An Advanced Dataset for Promoting Machine Learning Innovations in Realistic Chip Design Environment. Proceedings of the International Conference on Learning Representations (ICLR).

[17] Liang R., Agnesina A., Pradipta G., Chhabria V.A., Ren H. Invited Paper: CircuitOps: An ML Infrastructure Enabling Generative AI for VLSI Circuit Optimization. Proceedings of the IEEE/ACM International Conference on Computer-Aided Design (ICCAD).

[18] Dong Z., Cao W., Zhang M., Tao D., Chen Y., Zhang X. CktGNN: Circuit Graph Neural Network for Electronic Design Automation. Proceedings of the International Conference on Learning Representations (ICLR).

[19] Fang W., Lu Y., Liu S., Zhang Q., Xu C., Wills L.W., Zhang H., Xie Z. MasterRTL: A Pre-Synthesis PPA Estimation Framework for Any RTL Design. Proceedings of the IEEE/ACM International Conference on Computer-Aided Design (ICCAD).

[20] Fang W., Wang J., Lu Y., Liu S., Wu Y., Ma Y., Xie Z. (2025). A Survey of Circuit Foundation Model: Foundation AI Models for VLSI Circuit Design and EDA. arXiv.

[21] Ko Y., Burgstaller B. BEC: Bit-Level Static Analysis for Reliability against Soft Errors. Proceedings of the IEEE/ACM International Symposium on Code Generation and Optimization (CGO).

[22] Hari S.K.S., Tsai T., Stephenson M., Keckler S.W., Emer J.S. SASSIFI: An Architecture-Level Fault Injection Tool for GPU Application Resilience Evaluation. Proceedings of the IEEE International Symposium on Performance Analysis of Systems and Software (ISPASS).

[23] Sini J., Violante M., Tronci F. (2022). A Novel ISO 26262-Compliant Test Bench to Assess the Diagnostic Coverage of Software Hardening Techniques against Digital Components Random Hardware Failures. Electronics.

[24] Magliano E., Savino A., Di Carlo S. (2025). Real-Time Embedded System Fault Injector Framework for Micro-Architectural State Based Reliability Assessment. J. Electron. Test..

[25] OpenCores (2026). I2C Controller Core. https://opencores.org/projects/i2c.

[26] OpenCores (2026). SPI Master/Slave Interface. https://opencores.org/projects/spi_master_slave.

[27] OpenCores (2026). Versatile FIFO. https://opencores.org/projects/versatile_fifo.

[28] PULP Platform (2026). PULPino: Open-Source Microcontroller System Based on RISC-V. https://github.com/pulp-platform/pulpino.

[29] Rumelhart D.E., Hinton G.E., Williams R.J. (1986). Learning Representations by Back-Propagating Errors. Nature.

[30] Wu M., Yao Z., Ye L., Ge J., Zha J., Zhu Y. (2026). A Synergistic Framework Combining Explainable AI and Multiphysical Simulation for Uncovering Process–Structure Relationships in Mask Electrolyte Jet Machining. Adv. Eng. Inform..

